# Is the Present Peer-review System Sustainable?

**DOI:** 10.1007/s44402-026-00059-7

**Published:** 2026-03-13

**Authors:** Mark Rosenfield

**Affiliations:** State University of New York College of Optometry, New York, New York USA

Since 2021, the number of submissions to OPO has increased by ~85%, which means we now need 85% more reviewers than we did 5 years ago. As the current Editor-in-Chief, without question, the most difficult part of the job is trying to find individuals willing to review manuscripts. I understand the frustration. Doing a good review probably takes between 10 and 20 h of work, for which the reviewer will receive zero financial compensation. So why would anyone in their right mind be willing to undertake such a task?

I think the answer is simple, and yet very unsatisfying. As scientists, clinicians and educators, we need to tell the world about our work, so that advances in knowledge, methods of practice and treatment options can take place. But should anybody be able to promote a hypothesis or theory that has not gone through at least some level of evaluation prior to publication? I suspect most of us would agree that a gatekeeping process is necessary to preserve standards. Otherwise, we end up with a free-for-all, where anything can be proposed, irrespective of supporting data, also known in general as the Internet and specifically as social media. Indeed, I suspect that one of the reasons why the world is in its current state is the fact that everyone has a voice, irrespective of whether their comments are justified or supported by high-quality evidence. I make no apology for stating that the manuscripts in OPO are written by experts, to be read by current (and aspiring) experts. In recent years, experts have received considerable bad press, especially from politicians, and it has been proposed that an expert opinion carries no more weight than that of the layperson. I profoundly disagree. While experts can certainly be wrong, I still value their opinion more than the untrained individual with a loud voice, a bully pulpit or an online megaphone.

So what does all of this have to do with peer-review? We need high-quality journals so that science can continue to advance. If knowledge and treatment options stand still, then effectively, we are going backwards. Nobody wants medicine or optometry to be practiced the way they were carried out 100 years ago. There must be an evaluation process to determine what should, and perhaps even more importantly, should not be published. If you believe in that premise, then the current standard is the peer-reviewed journal. Evidently, it is not ideal, perfect or unfailing. We have all read papers and asked ourselves what the reviewers or editor was thinking when they accepted this manuscript for publication. But until we have anything better, then this is the benchmark, and for now at least, I believe it needs to survive.

I think it comes down to a relatively straightforward concept. If you want peer-reviewed journals to exist, so that you have places to publish your work, then you need to be willing to act as a peer reviewer. As an editor, I have learned which individuals always say no to review requests, some of whom are prolific authors. In some countries, the concept of acting as a peer reviewer does not seem to exist, so they always decline the invitation. I could keep sending these people manuscripts to evaluate, or even publish their names in the journal, but what would that achieve? Perhaps just a deformation lawsuit or two. It has been suggested that if they refuse to review, maybe we should refuse to publish their work. But that would mean keeping “blacklists”, and what if they were unable to review a manuscript for genuine reasons such as sickness, a conflict of interest, travel plans or simply receiving a paper that was not in their area of expertise. Therefore, I am not going there, at least for now.

Somehow, I have to appeal to your generosity and willingness to advance optometry, eye care and science in general. Is it an unrewarded and often frustrating exercise that carries no financial gain? Absolutely. But you will be improving knowledge and perhaps advancing the career of a young researcher. Therefore, all I can do is ask, cajole, beg or plead with you to help. This and other journals simply cannot exist without reviewers, and if colleagues are not willing to undertake the task, then the journals may cease to exist, giving us nowhere to publish our research findings. Maybe universities could be persuaded to give faculty more credit for reviewing papers, especially when it comes to decisions regarding tenure and promotion. After all, simply being asked to review a manuscript indicates a degree of recognition of being knowledgeable in that particular field. As long as academics are expected to publish in peer-reviewed journals, then they have a role to ensure that such journals continue to thrive.

And finally, one more request, and I realise that I am pushing my luck here, but if you do agree to act as a reviewer (which will be greatly appreciated), then please try and submit the review as close to the requested deadline as possible. If you need a brief extension, please just ask, and I will try to be as accommodating as possible. But also understand that the authors are very anxious to see their work in print. I personally have a manuscript that has been with a journal (not OPO) for close to 6 months without having received any feedback, and so I appreciate the frustration of having to wait a long time for a response. Additionally, if you do agree to review a manuscript and there is a delay, then please keep the editorial office updated on the status. As hard as it is to find reviewers, it is even more demanding when we do not know if a review is coming or not. Even if the report is going to be late, at least the editorial office will know whether we need to look for more reviewers. The worst-case scenario is when an individual agrees to evaluate a paper but fails to submit a report. That frustrates authors, editors and publishers alike, and causes even more delays because it is unclear what the next steps should be.

So, in short, please try to help out. Ask yourself whether you want peer-reviewed journals to survive. If the answer is yes, then you need to be willing to play a role in the process to ensure you have places to publish your work. But if you are not willing to review manuscripts, then perhaps you should consider whether it is ethical to expect others to evaluate your work. That may sound harsh, but I think the entire system has reached a tipping point and may not survive if the current rate of increase in submissions continues along its present trend. As the Editor-in-Chief of OPO, I will continue to seek reviewers for all the papers considered worthy of being evaluated, but please understand the difficulties when it often takes 10, 20 or even 30 requests to find referees. And to the people who always or nearly always say yes, on behalf of the authors, publishers and Editorial Board members, a very sincere and large thank you. You are making a huge contribution to the advancement of science and eye care.

## Data Availability

No datasets were generated or analysed during the current study.

